# Newest data on fluoroquinolone resistance mechanism of *Shigella flexneri* isolates in Jiangsu Province of China

**DOI:** 10.1186/s13756-017-0249-1

**Published:** 2017-09-19

**Authors:** Tingting Qin, Huimin Qian, Wenting Fan, Ping Ma, Lu Zhou, Chen Dong, Bing Gu, Xiang Huo

**Affiliations:** 1 Department of Laboratory Medicine, Affiliated Hospital of Xuzhou Medical University, Xuzhou, 221002 China; 2 Jiangsu Provincial Center for Disease Control and Prevention, Nanjing, 210009 China; 3 Medical Technology School, Xuzhou Medical University, Xuzhou, 221004 China

**Keywords:** *Shigella flexneri*, *gyrA*, Plasmid, *qnrS*, Fluoroquinolone resistance

## Abstract

**Background:**

To determine the prevalence, antimicrobial susceptibility patterns and related presence of mutations in quinolone resistance-determining region (QRDR) genes and plasmid-mediated quinolone resistance (PMQR) among *Shigella flexneri* isolates obtained from Jiangsu Province, China.

**Methods:**

A total of 400 *Shigella flexneri* clinical isolates collected during 2012–2015 were identified by biochemical and serological methods, and the antimicrobial susceptibility pattern was evaluated using the disc-diffusion method. PCR and DNA sequencing were accomplished to identify mutations in *gyrA*, *gyrB*, *parC* and *parE*, and the presence of *qnrA*, *qnrB*, *qnrC*, *qnrD*, *qnrS*, *qepA* and *aac(6′)-Ib-cr* genes were also detected.

**Results:**

Of all the *Shigella flexneri*, 75.8% were resistant to nalidixic acid, and 37.0% were categorized as norfloxacin resistant. Overall, 75.5% of isolates possessed *gyrA* mutations (Ser83Leu, Asp87Gly/Asn and His211Tyr), while 84.3% had *parC* mutations (Ser80Ile, Ala81Pho, Gln91His and Ser129Pro). The most prevalent point mutations in *gyrA* and *parC* were Ser83Leu (75.5%, 302/400) and Ser80Ile (74.5%, 298/400), relatively. Besides, the Gln517Arg alternation in *gyrB* was detected in 13 *S. flexneri* isolates and no mutations were identified in *parE*. PMQR determinations of *qnrB*, *qnrS* and *aac(6′)-Ib-cr* were detected among 16 strains (4.0%).

**Conclusions:**

The results presented here show that fluoroquinolone resistance in these clinical isolates result from mutations in chromosome, besides, despite the low prevalence of PMQR determinants in Jiangsu, it is essential to continue surveillance PMQR determinants in this area.

## Background

*Shigella*, a common cause of diarrheal disease worldwide, is a significant public health burden, particularly in sub-Saharan Africa and Asia [1]. In China, morbidity due to diarrhoea has decreased from 84 million in 1988 episodes to approximately 0.8–1.7 million in 2000 [2], despite that, diarrhoeal diseases remain sixth in the rank of death caused by infectious disease in China. Moreover, Walker CL et al. reported that China is among the 15 high-burden countries where nearly three-quarters of diarrhoea mortality concentrated in [3]. Symptoms caused by *Shigella* infection ranges from fever and fatigue to diarrhoea and/or dysentery, and frequent mucoid bloody stools, abdominal cramps and tenesmus occurred within hours to days [4].

Among the four species of *Shigella* (*Shigella flexneri*, *Shigella sonnei*, *Shigella boydii* and *Shigella dysenteriae*), *S. flexneri* is the predominant species in developing countries and the most frequent cause of bacterial dysentery [5]. Presently, fluoroquinolones emerged as the preferred agents for treatment of *Shigella* infection, however, the progressive increase in antimicrobial resistance further narrows the choice of effective antimicrobials. Around China, an increase in the occurrence of antimicrobial resistance have been reported, resistance rate to norfloxacin was 22.3% in a surveillance between 2005 and 2011 in Anhui Province [6]. In our previous study, the proportion of norfloxacin-resistant *S. flexneri* was as high as 36.8% in the period 2006–2011 in Jiangsu Province [7].

The development of fluoroquinolone resistant was found to be associated with several mechanisms that have been documented, primarily attributed to multiple mutations in the quinolone resistance-determining region (QRDR) of the genes encoding DNA gyrase (*gyrA* and *gyrB*) and topoisomerase IV (*parC* and *parE*) [8], additionally, plasmid-mediated quinolone resistance (PMQR) and efflux pump mediators mechanisms have been identified related to resistance [9].

In our previous survey of *S. flexneri* isolated from 2001 to 2011, fluoroquinolone resistance profiles and the presence of QRDR genes encoding resistance to fluoroquinolone have been described, in this study, we wanted to assess the level of antimicrobial resistance and fluoroquinolone resistance mechanisms including QRDR and PMQR genes of *S. flexneri* collected from patients between 2012 and 2015 in Jiangsu Province, China. Indeed, this is the first work to detect PMQR determinants in *Shigella flexneri* strains from Jiangsu Province.

## Methods

### Strains collection and examination

A total of 400 non-duplicate, consecutive clinical *S. flexneri* strains were collected from Jiangsu Provincial Center for Disease Control and Prevention between January 2012 and December 2015, and the isolates enrolled were isolated throughout Jiangsu Province. Jiangsu Province is a region with approximately 100 thousand square kilometers and a population of 75 million and 13 cities located in this area. The cities were Nanjing (*n* = 9), Suzhou (*n* = 44), Wuxi (*n* = 22), Zhenjiang (*n* = 20), Changzhou (*n* = 40), Nantong (*n* = 27), Taizhou (*n* = 60), Yangzhou (*n* = 18), Xuzhou (*n* = 33), Yancheng (*n* = 25), Lianyungang (*n* = 44), Huai’an (*n* = 26), and Suqian (*n* = 32).

Samples were cultured for *Shigella* by streaking diarrheal stools directly onto *Salmonella*–*Shigella* agar and then incubated at 37 °C for 16 to 24 h. After culture and screening, the isolates were identified using VITEK 2 system (bioMérieux, Marcy l’Etoile, France).

### Serotyping

Serologic identification was performed by slide agglutination with polyvalent somatic (O) antigen grouping sera, followed by testing with available monovalent antisera for specific identification of serotypes according to the manufacturer’s instructions (Denka Seiken, Japan).

### Drug susceptibility test

Antibiotic susceptibility of the *Shigella* isolates was tested using the Kirby-Bauer method on Muller-Hinton agar plates for nalidixic acid (NAL) and norfloxacin (NOR) (Oxoid, Hampshire, UK), results were interpreted according to Clinical and Laboratory Standards Institute (CLSI) performance standards [10]. A control strain of *Escherichia coli* (ATCC 25922) used for antibiotic susceptibility testing quality control.

### Detection of point mutations in *gyrA*, *gyrB*, *parC*, and *parE*

Preparation of bacterial DNA templates and PCR amplification of the quinolone resistance-determining regions of *gyrA*, *gyrB*, *parC* and *parE* were performed using primers and conditions as previously described (Table 1) [11–13]. The presence of the target genes was confirmed through 1.5% agarose gel electrophoresis. Subsequently, the amplification products of QRDR genes were purified, then, DNA sequencing was performed using primers (Genewiz, Suzhou, China). The sequence data were analyzed by comparison with sequences in GenBank.

**Table 1:** Primes used in this study

Gene	Sequence (5′–3′)	Product size (bp)	Annealing temperature (°C)
*gyrA*	F: TACACCGGTCAACATTGAGG R: TTAATGATTGCCGCCGTCGG	648	60
*gyrB*	F: CAAACTGGCGGACTGTCAGG R: TTCCGGCATCTGACGATAGA	345	60
*parC*	F: GTCTGAACTGGGCCTGAATGC R: AGCAGCTCGGAATATTTCGACAA	248	60
*parE*	F: ATGCGTGCGGCTAAAAAAGTG R: TCGTCGCTGTCAGGATCGATAC	289	60
*qnrA*	F: ATTTCTCACGCCAGGATTTG R: GATCGGCAAAGGTTAGGTCA	515	50
*qnrB*	F: GATCGTGAAAGCCAGAAAGG R: ACGATGCCTGGTAGTTGTCC	468	54
*qnrC*	F: GGGTTGTACATTTATTGAATC R: TCCACTTTACGAGGTTCT	446	50
*qnrD*	F: CGAGATCAATTTACGGGGAATA R: AACAAGCTGAAGCGCCTG	581	50
*qnrS*	F: ACGACATTCGTCAACTGCAA R: TAAATTGGCACCCTGTAGGC	416	55
*aac(6′)-Ib-cr*	F: GCAACGCAAAAACAAAGTTAGG R: GTGTTTGAACCATGTACA	560	55
*qepA*	F: GCAGGTCCAGCAGCGGGTAG R: CTTCCTGCCCGAGTATCGTG	217	60

### Detection of PMQR

Besides, all isolates were screened for plasmid-mediated quinolone resistance (PMQR) genes (*qnrA*, *qnrB*, *qnrC*, *qnrD*, *qnrS*, *qepA* and *aac(6′)-Ib-cr*) as described in previous reports (Table 1) [14–18]. Next, the amplified PCR products were identified by agarose gel electrophoresis and the positive products were purified and sequenced (Genewiz, Suzhou, China) to confirm the presence of PMQR genes.

### Statistical analysis

For analysis of resistance patterns and mutation characteristics, statistical comparisons were performed using the chi-Square test. The differences among the groups were considered to be statistically significant at *P* < 0.05.

## Results

### Bacterial isolation

**Fig. 1:**
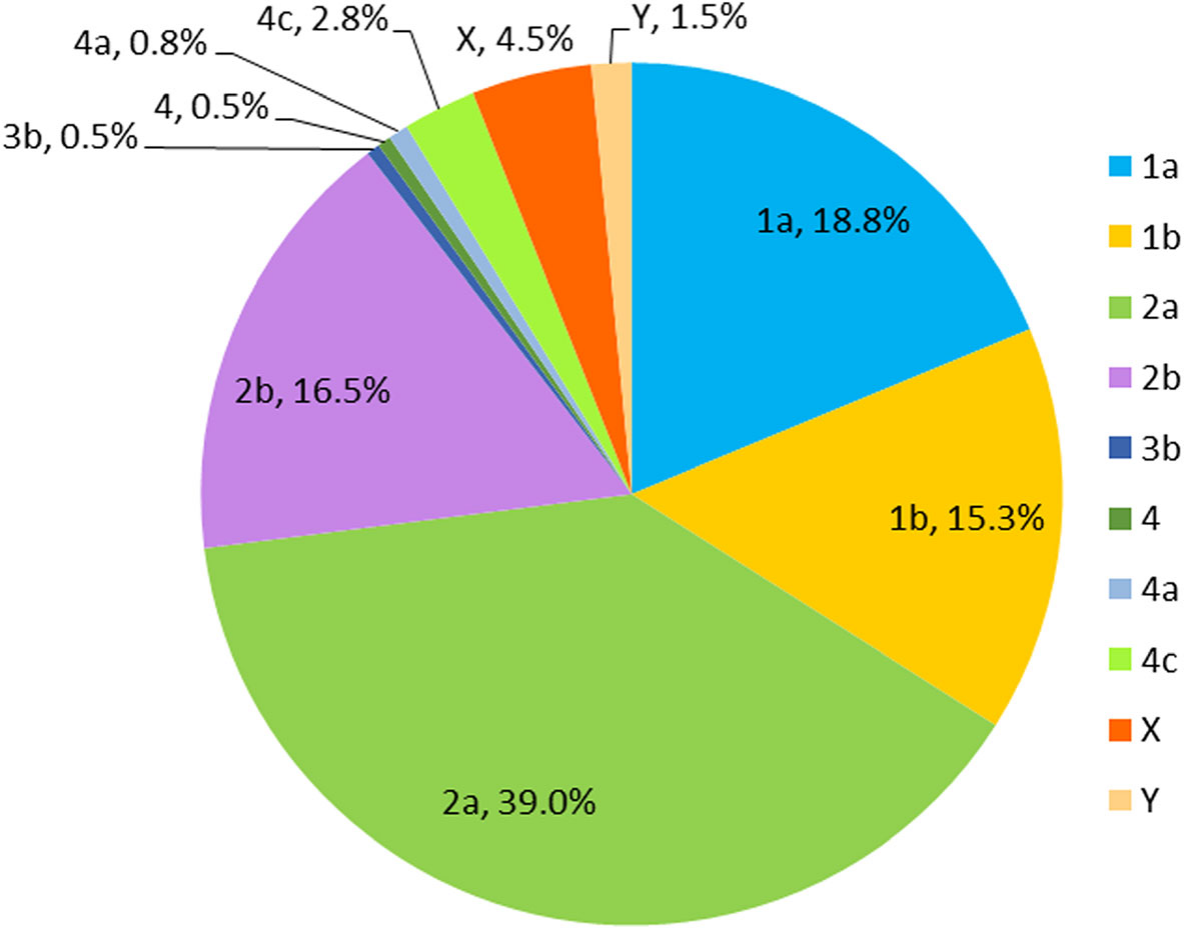
Serotypes of the *Shigella flexneri* strains

A number of 400 *S. flexneri* isolates were isolated and identified from patients over the period from 2012 to 2015 in Jiangsu Province of China. The number of isolated strains of the four years accounted for 120, 114, 85 and 81, relatively, showing a decreasing trend. Among these clinical *S. flexneri* isolates, 2a (*n* = 156, 39.0%) was the most prevalent serotype, followed by 1a (*n* = 75, 18.8%), 2b (*n* = 66, 16.5%), 1b (*n* = 61, 15.3%) and X (*n* = 18, 39.0%). Other serotypes including 3b, 4, 4a, 4c and Y were also detected (Fig. 1).

### Fluoroquinolone susceptibility testing

Of the 400 *S. flexneri* isolates, 75.8% (303) were resistant to nalidixic acid, and 37.0% (148) were categorized as norfloxacin resistant. During the four years, resistance rate of nalidixic acid fluctuated between 70.0% and 80.0% and no clear changing trend was observed. A comparative analysis of the resistance against norfloxacin performed illustrated that the proportion of norfloxacin resistant isolates between 2014 (36/85) and 2015 (33/81) was notably higher than that isolated in 2012 (41/120) and 2013 (38/114) (*P* < 0.05). Fluoroquinolone susceptibility profiles of the isolates also varied between serotypes. Serotype 1a showed the highest resistance rate to both nalidixic acid and norfloxacin, with resistance rates of 88.0% and 46.7%, respectively, and the proportions were closely followed by serotype 2a of 85.9% and 45.5%. Most noticeably was that all the *S. flexneri* isolates that identified as serotype 4, 4a, 4c, X and Y were nalidixic acid resistant and almost half of the serotype 4c isolates were resistant to norfloxacin.

### Mutations in DNA gyrase and topoisomerase IV genes

#### Mutations in *gyrA* gene

DNA sequencing results showed that 75.5% (302/400) of isolates possessed *gyrA* mutations, and all these isolates were detected with Ser83Leu. Most of the *S. flexneri* isolates (295/302) were detected with two or more mutations in *gyrA*, 229 isolates displayed Ser83Leu, Asp87Gly/Asn and His211Tyr simultaneously, 66 isolates displayed both Ser83Leu and His211Tyr. Additionally, the mutation of Ser83Leu and His211Tyr took up a stable share during the four years while the association between time distribution and amino acid substitution at codon 87 in the *parC* gene was statistically significant (*P* < 0.05) (Table 2). A significant annually decrease in the substitution of Asp-87 to Gly was seen (40.8% in 2012 to 13.6% in 2015) whereas the mutation rate of Asp-87 to Asn remained stable in the first two years and fluctuated largely in 2014 (Table2).

**Table 2:** Mutations in *gyrA*, *gyrB* and *parC* genes in *Shigella flexneri* strains in the present study

Mutations	2012 (*n* = 120)	2013 (*n* = 114)	2014 (*n* = 85)	2015 (*n* = 81)	Total (*n* = 400)
Ser83Leu	79.2 (95)	75.4 (86)	75.3 (64)	70.4 (57)	75.5 (302)
Asp87Asn/ Gly	65.0 (78)	50.9 (58)	61.2 (52)	50.6 (41)	57.3 (229)
Asp87Asn	24.2 (29)	24.6 (28)	41.2 (35)	37.0 (30)	30.5 (122)
Asp87 Gly	40.8 (49)	26.3 (30)	20.1 (17)	13.6 (11)	26.8 (107)
His211Tyr	75.0 (90)	74.6 (85)	75.3 (64)	69.1 (56)	73.8 (295)
Gln517Arg	5.8 (7)	3.5 (4)	2.5 (2)	0.0 (0)	3.3 (13)
Ser80Ile	77.5 (93)	75.4 (86)	75.3 (64)	67.9 (55)	74.5 (298)
Ser129Pro	33.3 (40)	41.2 (47)	52.9 (45)	24.7 (20)	38.0 (152)

#### Mutations in *parC* gene

For the *parC* gene, 84.3% (337/400) had mutations, 298 and 152 isolates carried substitution at codon Ser80 and Ser129, relatively. Of those strains, 113 showed double mutations of Ser80Ile and Ser129Pro, remarkably, these 113 isolates were also observed with mutations at codon Ser83 and His211 in *gyrA*. Addtionally, Gln91His substitution in *parC* was detected in 2 isolates from 2012 and one of the two also revealed the amino acid substitution Ala81Pho in *parC*. During the four years, the mutation rate of Ser80Ile ranged between 67.9% and 77.5%. In contrast, the mutation rate of Ser129Pro kept rising from 2012 to 2014, and then decreased to 24.7% in 2015 (Table 2).

#### Mutation in *gyrB* and *parE* genes

Among the *S. flexneri* isolates, 3.3% (13/400) were detected with amino acid substitution at Gln-517 to Arg in *gyrB* and no mutations were identified in *parE* (Table 2). Furthermore, 10 out of the 13 alternation in *gyrB* gene showed another four point mutations in either *gyrA* or *parC*.

### Mutations and fluoroquinolone susceptibility

There were 94.4% (286/303) of the nalidixic acid resistant *S. flexneri* carried Ser83Leu mutation (Table 3). Attentionally, obvious difference of Asp87Gly/Asn was observed between norfloxacin susceptible and resistant isolates (*P* < 0.05), Asp87Gly/Asn occurred more often among norfloxacin resistant *S. flexneri*. Another mutation in *gyrA*, the substitution His-211 to Tyr among fluoroquinolone susceptible was much lower than that in fluoroquinolone resistant strains. The Ser129Pro in *parC* was identified as no difference in fluoroquinolone susceptible and resistant *S. flexneri* according to statistical analysis (*P* > 0.05). A total of 81.8% (121/148) of the norfloxacin resistant *S. flexneri* contained at least three amino acid substitutions of Ser83Leu and Asp87Gly/Asn in *gyrA* and Ser80Ile in *parC*. Surprisingly, 11.8% (11/93) of the fluoroquinolone susceptible isolates also carried the three common mutations in *gyrA* and *parC*.

**Table 3:** Mutations and fluoroquinolone susceptibility of *Shigella flexneri* strains

Point mutations	NAL-S & NOR-S strains^a^ (*n* = 93)	NAL-R strains^b^ (*n* = 303)	NAL-R & NOR-S strains (*n* = 84)	NOR-R strains (*n* = 148)
No mutation	46 (49.5)	10 (3.3)	5 (6.0)	5 (3.4)
*GyrA*-Ser83Leu	14 (15.1)	286 (94.4)	77 (91.7)	138 (93.2)
*GyrA*-Asp87Asn/Gly	11 (11.8)	217 (71.6)	28 (33.3)	122 (82.4)
*GyrA*-His211Tyr	13 (13.9)	280 (92.4)	74 (88.1)	135 (91.2)
*GyrB*-Gln517Arg	3 (3.2)	10 (3.3)	3 (3.6)	3 (2.0)
*ParC*-Ser80Ile	15 (16.1)	281 (92.7)	75 (89.3)	135 (91.2)
*ParC*-Ser129Pro	35 (37.6)	116 (38.3)	37 (44.0)	48 (32.4)
*GyrA*-Ser83Leu & *ParC*-Ser80Ile	13 (13.9)	280 (92.4)	75 (89.3)	134 (90.5)
*GyrA*-Ser83Leu & *ParC*-Ser80Ile & *GyrA*-Asp87Asn/Gly	11 (11.8)	216 (71.3)	28 (33.3)	121 (81.8)

*NAL-S* nalidixic acid susceptible, *NAL-R* nalidixic acid resistant, *NOR-S* norfloxacin susceptible, *NOR-R* norfloxacin resistant. ^a^NAL-S & NOR-S strains, besides these strains, there were 3 strains showed NAL-S & NOR-I (intermediate resistant/susceptible) and 1 NAL-I & NOR-S strain. ^b^NAL-R strains, among these strains, there were 71 NAL-R & NOR-I strains, which were not analysed in the table. The isolates that showed intermediate susceptible/resistant to NAL/NOR were not listed in detail in this table

### Mutations and serotypes

Also, the significant difference between serotypes and Asp-87 mutation in *parC* was observed. There were almost no mutations in *S. flexneri* serotype 1b (Table 4). Among the common serotypes, the proportion of substitution Asp-87 to Asn in serotype 2a was much more (64.1%) higher than that in other serotypes (*P* < 0.05). In contrast, the change of Asp-87 to Gly was highly prevalent in serotype 2b (59.1%) and 1a (52.0%), however, presenting a decreasing trend during 2012–2015.

**Table 4:** The Asp-87 mutation in *gyrA* in the four predominate *Shigella flexneri* serotypes

Serotypes	Mutation	Years				Total
		2012 (%^a^)	2013 (%^b^)	2014 (%^c^)	2015 (%^d^)	
2a (*n* = 156)	*gyrA*-Asp87Asn	59.6 (28/47)	53.7 (22/41)	63.6 (21/33)	82.9 (29/35)	64.1 (100/156)
	*gyrA*-Asp87Gly	23.4 (11/47)	9.8 (4/41)	15.2 (5/33)	14.3 (5/35)	16.0 (25/156)
1a (*n* = 75)	*gyrA*-Asp87Asn	0.0 (0/25)	10.7 (3/28)	53.8 (7/13)	0.0 (0/9)	13.3 (10/75)
	*gyrA*-Asp87Gly	88.0 (22/25)	39.3 (11/28)	23.1 (3/13)	33.3 (3/9)	52.0 (39/75)
2b (*n* = 66)	*gyrA*-Asp87Asn	0.0 (0/21)	10.5 (2/19)	8.3 (1/12)	7.1 (1/14)	6.1 (4/66)
	*gyrA*-Asp87Gly	76.2 (16/21)	68.4 (13/19)	58.3 (7/12)	21.4 (3/14)	59.1 (39/66)
1b (*n* = 61)	*gyrA*-Asp87Asn	0.0 (0/20)	0.0 (0/10)	0.0 (0/12)	0.0 (0/19)	0.0 (0/61)
	*gyrA*-Asp87Gly	0.0 (0/20)	0.0 (0/10)	0.0 (0/12)	0.0 (0/19)	0.0 (0/61)

^a^% = Mutation strains/number of different serotypes in 2012

^b^% = Mutation strains/number of different serotypes in 2013

^c^% = Mutation strains/number of different serotypes in 2014

^d^% = Mutation strains/number of different serotypes in 2015

### Characterization of PMQR genes

PMQR determinants were detected alone or in combination in 4.0% (16 of 400) of isolates, *qnrB*, *qnrS* and *aac(6′)-Ib-cr* were present in 0.3% (1/400), 2.0% (8 /400) and 2.0% (8/400) of those isolates, respectively (Table 5), while the *qnrA*, *qnrC*, *qnrD* and *qepA* genes were not detected. A *qnrS* positive isolate was detected in combination with *aac(6′)-Ib-cr*. Except for the *qnrB* positive isolate without any mutation, 15 of the 16 PMQR determinants positive isolates had at least two mutations of Ser83Leu in *gyrA* and Ser80Ile in *parC* gene. Of all the PMQR determinants’ positive isolates, 87.5% (14/16) were resistant to nalidixic acid and 56.3% (9/16) of them were resistant to norfloxacin.

**Table 5:** Characteristics of the PMQR determinants positive S*higella flexneri* isolates

Isolate number	Serotype	Year of isolation	City	Antibiotic susceptibility		Target gene mutations						PMQR determinants
				NAL	NOR	*gyrA*			*gyrB*	*parC*		
F12002	3b	2012	Nantong	R	R							*qnrB*
F12019	2b	2012	Yangzhou	R	I	Ser83Leu	Asp87Gly	His211Tyr		Ser80Ile		*aac(6′)-Ib-cr*
F12037	2a	2012	Zhenjiang	S	I	Ser83Leu		His211Tyr		Ser80Ile		*aac(6′)-Ib-cr*
F12066	4c	2012	Xuzhou	R	R	Ser83Leu		His211Tyr		Ser80Ile		*aac(6′)-Ib-cr*
F12099	2b	2012	Yangzhou	R	I	Ser83Leu	Asp87Gly	His211Tyr		Ser80Ile	Ser129Pro	*aac(6′)-Ib-cr*
F12149	4c	2012	Xuzhou	R	S	Ser83Leu		His211Tyr		Ser80Ile	Ser129Pro	*aac(6′)-Ib-cr*
F12150	2a	2012	Xuzhou	R	I	Ser83Leu		His211Tyr		Ser80Ile	Ser129Pro	*aac(6′)-Ib-cr*
F13055	4c	2013	Changzhou	R	R	Ser83Leu		His211Tyr		Ser80Ile	Ser129Pro	*qnrS*
F13140	1a	2013	Yangzhou	S	S	Ser83Leu		His211Tyr	Gln517Arg	Ser80Ile		*aac(6′)-Ib-cr*
F14054	2b	2014	Suqian	R	I	Ser83Leu	Asp87Asn	His211Tyr		Ser80Ile		*aac(6′)-Ib-cr*, *qnrS*
F15099	2a	2015	Taizhou	R	R	Ser83Leu	Asp87Asn	His211Tyr		Ser80Ile		*qnrS*
F15100	2a	2015	Taizhou	R	R	Ser83Leu	Asp87Asn	His211Tyr		Ser80Ile		*qnrS*
F15101	2a	2015	Taizhou	R	R	Ser83Leu	Asp87Asn	His211Tyr		Ser80Ile		*qnrS*
F15105	2a	2015	Taizhou	R	R	Ser83Leu	Asp87Asn	His211Tyr		Ser80Ile		*qnrS*
F15106	2a	2015	Taizhou	R	R	Ser83Leu	Asp87Asn	His211Tyr		Ser80Ile	Ser129Pro	*qnrS*
F15107	2a	2015	Taizhou	R	R	Ser83Leu	Asp87Asn	His211Tyr		Ser80Ile		*qnrS*

*NAL* nalidixic acid, *NOR* norfloxacin; antibiotic susceptibility, *S* susceptible, *I* intermediate resistant/susceptible, *R* resistant

## Discussion

Our data documented an ongoing decreasing trend of *S. flexneri* from 2012 to 2015, which is reflective of a current shift in the epidemiologic distribution of this specie in China in recent years [19, 20]. The same tendency of *S. flexneri* observed not only in China but also in some other countries like Bangladesh and Thailand [21, 22]. However, due to the unbalanced socioeconomic conditions, the distribution of *Shigella* infection species varies within the same country, a previous study indicated that lower rates of *S. flexneri* cases were observed in East, North and Northeast China, as compared to those in Middle, South, Northwest and Southwest China [19]. A retrospective study we conducted before have reported that 16 serotypes was observed between 2001 and 2011 in Jiangsu Province, with serovars 2a, 2b and 1a being the most common serotypes [7], in this study, we found that the top three most common *S. flexneri* serotypes were consistent with that years ago. Overall, both serotypes 4c and X accounted for a proportion in the last 15 years [7]. All this illustrated that the predominant serotypes 2a, 2b and 1a remained important serotype, and distribution of some other serotypes fluctuated over the years. However, what’s worrisome is that the only vaccine currently in use against *S. flexneri* in China is a live serotype 2a vaccine [23], hence a broad-spectrum *Shigella* vaccine that protect against all the *S. flexneri* serotypes/subserotypes is necessary [5].

At present, the occurrence and prevalence of fluoroquinolone-resistant *S. flexneri* poses a great challenge for the effective treatment of shigellosis. Fluoroquinolone-resistant *Shigella* has been explicitly listed as one of the top concerns in the current international focus on antimicrobial resistance [24]. In this paper, we revealed a high rate of resistance to nalidixic acid (75.8%), lower than that in 2006–2011 [7]. Additionally, we described here the worrisome fact that 37.0% of the *S. flexneri* isolates conferred resistance to norfloxacin. The present data showed a slight increasing of norfloxacin resistance rate, and, in contrast, a decreasing tendency of nalidixic acid resistance patterns, consistent with previous reports [7, 25].

Fluoroquinolone susceptibility profiles of *S. flexneri* varied between geographical locations. Nalidixic acid and norfloxacin resistance was documented in 100.0% and 22.3% of *S. flexneri* isolates in Anhui Province from 2005 to 2011 [6]. In Shanghai, all the *S. flexneri* showed nalidixic acid resistance [23]. In contrary, only 14.0% of *S. flexneri* was resistant to nalidixic acid and 8.4% to ciprofloxacin in Switzerland during 2004–2014 [26]. Overall, the results indicated the emerging problem that an increasing number of *S. flexneri* isolates have lost the natural susceptibility to fluoroquinolones. The worsening situation may be attributed to inappropriate prescription, easy access to antimicrobials and lack of local surveillance of antimicrobial resistance [25].

Besides, we observed the resistance patterns between serotypes. Serotypes 1a and 2a were demonstrated with higher rates of resistance to fluoroquinolones, which was consistent with that reported before [25]. Hence, antibiotic susceptibility testing performed at the start of an outbreak is highly recommended, and more prudent selection and use of fluoroquinolones are warranted. Moreover, it is of great importance to conduct continuous and strong surveillance of fluoroquinolone resistance of *Shigella* in local areas for periodic updating of the local antibiograms.

This study revealed that substitutions within *gyrA* at Ser83 and His211 and Ser80 within *parC* were most frequently observed in the study years. Different with reported studies [27, 28], the presence of His211Tyr in *gyrA*, was of high prevalence among fluoroquinolone resistant *S. flexneri* isolates, and all the mutation was found with the concomitant presence of Ser83Leu mutation, the findings was similar to a study on *S. flexneri* serotype 4 s [29], indicating that His211Tyr have been found in several provinces around China. Previous study have shown that mutations in the primary target genes of fluoroquinolones in *Shigella* can lead to development of decreased susceptibility to fluoroquinolones [9], thus complicating the treatment of infections. Typically, resistance to nalidixic acid resulted mainly from a single amino acid substitution at either position 83 or position 87 of the *gyrA* gene [30]. Among the norfloxacin resistant isolates, 91.2% showed the presence of at least three substitutions in *gyrA* and *parC*, and 81.8% presented the three common mutations Ser83Leu, Asp87Gly/Asn and Ser80Ile. Our results are consistent with the previous report that the presence of additional mutations in *gyrA* at position 87 and *parC* mutation at either position 80 contributed to high-level fluoroquinolone resistance [9]. Unfortunately, the fluoroquinolone susceptible isolates also carried mutations within *gyrA* and *parC*, leading to difficulty in clinical treatment.

Moreover, an interesting finding in our study was that the amino acid substitution at codon 87 in the *parC* gene varied significantly with serotypes and presented yearly variations. The mutation rate of Asp-87 to Gly decreased annually during the four years, and Asp-87 to Gly was highly prevalent in serotype 2b and 1a while substitution of Asp-87 to Asn in serotype 2a was much more prevalent. However, limited reports on epidemiology of serotypes and mutations were published. Furthermore, less common, the substitution Gln91His and Ala81Pho were observed in *parC* and novel point mutation Gln517Arg in *gyrB* gene was detected, which is worthy of notice.

There is scarce information on the prevalence of PMQR in Jiangsu Province while reports in other areas were common [27, 31–33]. The overall prevalence of PMQR in *S. flexneri* in the present study was 4.0%, lower than that in Anhui and Henan, rates of 9.6% and 8.2%, respectively [27, 31]. *aac(6′)-Ib-cr* and *qnrS* were the most frequent PMQR determinants detected among *S. flexneri*. This agrees with previous reports that *qnrS* and *aac(6′)-Ib-cr* were the predominant PMQR determinants across two provinces located in Eastern China [31, 32]. Additionally, one of the *aac(6′)-Ib-cr* positive isolates co-harbored the *qnrS* gene. The frequency of *qnrB* in this study was similar to the previous report in China [28, 31]. Almost all of the PMQR-positive isolates carried uniform mutations Ser83Leu in *gyrA* and Ser80Ile in *parC*, which may attribute to the promotion mutations within the QRDR, and result in resistance to fluoroquinolones. Furthermore, the *qnrS*-positive isolates in the present study demonstrated the report that the presence of *gyrA* mutation(s) and/or the *qnrS* gene dramatically increased fluoroquinolone resistance for *S. flexneri* [34]. Given that *Shigella* harboring the *qnrS* gene are able to replicate efficiently in high concentrations of fluoroquinolones [34], the potential dissemination of fluoroquinolone resistance is evident. Seven of the PMQR-positive isolates were not norfloxacin resistant, which is in accordance with a previous published study in Switzerland [26], illustrating the potential for development of resistance in susceptible strains. Therefore, therapeutic efficiency of fluoroquinolones may be decreased [26, 33].

Our study had several limitations. First, Collection of *S. flexneri* may not a comprehensive of *Shigella* infection in Jiangsu Province because some people with shigellosis may not go to the hospital for medical care and diagnosis. Second, the *qnr* alleles of *qnrB* and *qnrS* positive *S. flexneri* strains in this study are not detected, which make it difficult to better evaluate the prevalent trends of PMQR determinants in this area. Moreover, whether the His211Tyr in *gyrA*, Gln91His, Ala81Pho and Ser129Pro in *parC* and novel point mutation Gln517Arg in *gyrB* facilitate fluoroquinolone resistance is unknown and will be further studied in our next step.

## Conclusion

Our study provides a baseline information on the prevalence in *S. flexneri* isolates of fluoroquinolone resistance and associated mechanisms including mutations in QRDR and PMQR determinants. Particularly, the significant finding of our study is the prevalence and distribution of PMQR genes in *Shigella* isolates in Jiangsu, China. Therefore, continuing active surveillance of antimicrobial resistance in *Shigella* is necessary and antimicrobial treatment must be updated regularly based on surveillance results.

## Abbreviations

CLSI: Clinical and Laboratory Standards Institute
NAL: Nalidixic acid
NOR: Norfloxacin
PMQR: Plasmid-mediated quinolone resistance
QRDR: Quinolone resistance-determining region
